# Reduced portal vein blood flow velocity in acute fatty liver of pregnancy

**DOI:** 10.3389/fmed.2024.1506340

**Published:** 2024-12-23

**Authors:** Yang Zhao, Er-ke Zhang, Dan-Hua Li, Yu-Jing Zhou, Hui Jiang, Xiao-Chao Jia, Chuan Qin

**Affiliations:** ^1^Department of Medical Ultrasound, Jinshan Hospital of Fudan University, Shanghai, China; ^2^Department of Medical Ultrasound, The Central Hospital of Karamay, Karamay, China

**Keywords:** acute fatty liver of pregnancy, portal vein flow velocity, liver function, Doppler ultrasonography, prenatal care

## Abstract

**Purpose:**

Acute fatty liver of pregnancy (AFLP) is a severe complication that can occur in the third trimester or immediately postpartum, characterized by rapid hepatic failure. This study aims to explore the changes in portal vein blood flow velocity and liver function during pregnancy, which may assist in the early diagnosis and management of AFLP.

**Methods:**

This longitudinal study was conducted at a tertiary healthcare center with participants recruited from routine antenatal check-ups. The study included healthy women aged 20–40 years with singleton pregnancies. Doppler ultrasonography was used to assess portal vein flow and velocity, complemented by standard laboratory tests to monitor liver function. A nomogram model integrating the clinical features and Doppler ultrasonography parameters was constructed using logistic regression analysis for differentiating AFLP from normal controls.

**Results:**

A total of 135 women were enrolled, divided into control and AFLP groups. The AFLP group demonstrated a significant reduction in portal vein flow velocity and alterations in liver dimensions. Laboratory tests indicated elevated liver enzymes and altered lipid profiles in the AFLP group. Statistical analysis showed that portal vein flow velocity could be a sensitive marker for predicting liver dysfunction in AFLP. The area under the receiver operating characteristic (ROC) curves (AUC) of the nomogram model were 0.88 (95% confidence interval [CI]: 0.82–0.95) with specificity, sensitivity, negative predictive value, and positive predictive value of 67.8, 95.0, 95.0, and 67.8, respectively, in the training cohort and 0.86 (95% CI: 0.72–0.98) and 86.9, 75.0, 83.3, and 80.0 in the test cohort, respectively. The calibration curves demonstrated good agreement between the predicted and observed probability for predicting AFLP.

**Conclusion:**

The study underscores the potential of using portal vein flow velocity as an early diagnostic marker for AFLP in pregnant women. Further research is required to establish standardized diagnostic thresholds for portal vein flow measurements.

## 1 Introduction

Acute fatty liver of pregnancy (AFLP) is a rare but serious complication occurring in the third trimester of pregnancy or immediately postpartum. AFLP is characterized by microvesicular fatty infiltration of hepatocytes, which can lead to hepatic failure. This condition poses significant risks to both maternal and fetal health. Early diagnosis and prompt intervention are crucial but challenging, given the non-specific nature of initial symptoms.

Pregnancy induces a unique physiological state characterized by alterations in hepatic venous pulsatility and portal venous velocity, which is crucial for maintaining adequate blood flow to vital organs and ensuring fetal development (1). These changes are typically transient in permanency (2). Portal vein flow velocity is crucial in understanding liver function, particularly in pathological states of pregnancy-specific liver diseases, such as pre-eclampsia, eclampsia, AFLP, and HELLP syndrome, a serious condition characterized by hemolysis, elevated liver enzymes, and low platelets, are both severe complications of pregnancy (3).

Previous studies have focused on understanding these physiological changes using Doppler ultrasonography. A study reported that maximum flow velocity of the portal vein was higher in the first trimester than in the second and third trimesters (4). Another study found that maximum flow velocities of both the right hepatic vein and main portal veins were lower in the third trimester compared to the second trimester and the control group (5). Furthermore, a case reported that the mean maximum portal blood-flow velocity obtained at the umbilical-point of the left portal vein decreased during the acute phase of AFLP and returned to the normal range (6). The incidence of biphasic pattern was prominent in the second trimester, whereas the monophasic pattern was prominent in the third trimester, suggesting these as sensitive parameters of physiological adaptations related to pregnancy. In the hepatic vein, the triphasic waveform was prominent in the first trimester, changing to biphasic in the later trimesters. These findings indicate that flow pattern changes of the hepatic vein and flow velocity changes of the portal vein can be sensitive parameters indicating physiological alterations related to pregnancy.

In cases of AFLP, liver dysfunction can lead to altered hemodynamics, which may be reflected in the measurements of portal vein blood flow velocity. Research suggests that reductions in portal vein blood flow velocity and alterations in the waveform might indicate hepatic dysfunction (7). This could potentially assist in the early diagnosis of AFLP, especially when coupled with clinical findings and liver function tests (8). The potential of portal vein blood flow measurements in predicting and diagnosing AFLP remains underexplored. Current evidence, though preliminary, suggests that significant deviations from normal portal blood flow patterns could serve as early indicators of hepatic complications in pregnancy. Further studies are required to establish standardized thresholds for flow measurements that could be integrated into clinical practice.

We hypothesized that changes in portal vein blood flow velocity during pregnancy could serve as an early indicator of AFLP. To conduct a longitudinal study assessing AFLP and portal vein flow and velocity throughout pregnancy and the postpartum period. The exploration of portal vein blood flow velocity would be used as a potential marker for AFLP in pregnant women.

## 2 Materials and methods

### 2.1 Ethical considerations

This study was approved by the Institutional Review Board of Jinshan Hospital (Number: JIEC2023-S99). The study was conducted in accordance with the Declaration of Helsinki and relevant ethical guidelines for research involving human subjects. Written informed consent was obtained from all participants before the collection of any samples.

### 2.2 Study design and setting

This study was conducted at a local healthcare center with a well-established obstetrics and gynecology department, including healthy women aged 20–40 years with singleton pregnancies. Data collection was conducted from 10 January 2024 to 1 October 2024.

### 2.3 Participants

Pregnant women attending routine antenatal check-ups were recruited. Inclusion criteria include healthy women with singleton pregnancies and gestational age confirmed by ultrasound. Exclusion criteria include pre-existing liver diseases, multiple pregnancies, significant obstetric complications (e.g., preeclampsia), known chronic liver diseases unrelated to pregnancy, history of alcohol or drug abuse, and contraindications for ultrasound examination. The pregnant women were divided into two groups based on liver disease status (AFLP and Control). Finally 135 subjects were enrolled. The whole dataset was divide randomly in a training cohort and a test cohort (ratio of 7:3) for further analysis.

### 2.4 Laboratory tests

Laboratory tests including alanine aminotransferase, aspartate aminotransferase, total bile acids, red blood cell count, platelet count, glycated hemoglobin, triglycerides, total cholesterol, low-density lipoprotein cholesterol, high-density lipoprotein cholesterol, urea nitrogen, creatinine, uric acid, prothrombin time, albumin, total bilirubin, glucose, c-reactive protein were recorded.

### 2.5 Doppler ultrasonography portal vein flow and velocity measurement

Doppler ultrasonography was used to assess portal vein flow and velocity at the same intervals. Doppler ultrasound examinations was conducted using a high-resolution ultrasound machine with a 3.5 MHz curvilinear transducer. Liver function tests was analyzed using standard biochemical methods. Doppler ultrasound was performed to assess portal vein flow and velocity at predefined pregnancy stages. The measurements were standardized by using Doppler ultrasonography with a consistent technique across all participants following established protocols for Doppler assessment. The same experienced sonographer performed all scans to ensure uniformity in positioning and angle of insonation.

### 2.6 Nomogram model construction, discrimination, calibration, and validation

Multivariate logistic regression analysis was performed to select the independent predictors of clinical features and Doppler ultrasonography parameters for predicting ALFP. A nomogram model integrating the clinical features and Doppler ultrasonography parameters was constructed using logistic regression analysis. The goodness of fit of the nomogram was evaluated by calibration curves and the Hosmer-Lemeshow tests in the training and test cohorts. The predictive performances of the nomogram model were assessed using the AUC in both cohorts.

### 2.7 Statistical analysis

R (version 4.4.0)^1^ was used for statistical analysis. Descriptive statistics was used to summarize participant characteristics. Changes in liver function tests and Doppler measurements across pregnancy stages were analyzed using repeated measures ANOVA. Correlation analysis was used for exploring associations between liver function tests changes and Doppler findings. Descriptive statistics was used to summarize demographic, ultrasound, and liver function tests data. The normality of data was checked. Group comparisons was made using the *t*-test or Mann-Whitney U test, depending on data distribution. To explore the relationship between liver function and portal vein parameters, Pearson or Spearman correlation was used. Multiple regression analysis was employed to control for confounding variables. A *p*-value < 0.05 was considered statistically significant.

## 3 Results

**FIGURE 1:**
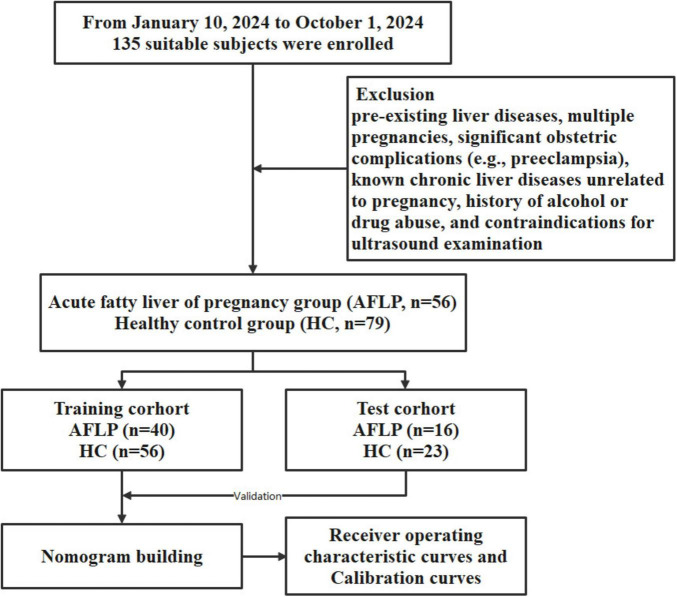
Flowchart of participant enrollment and study design. A total of 135 suitable subjects were enrolled in the study. Participants were divided into two groups: the acute fatty liver of pregnancy (AFLP) group (*n* = 56) and the healthy control (HC) group (*n* = 79). The subjects were further randomized into training and test cohorts, with the training cohort consisting of 40 AFLP and 56 HC subjects and the test cohort consisting of 16 AFLP and 23 HC subjects. The training cohort data were used for building a nomogram model, which was validated using the test cohort. The predictive performance of the model was evaluated using receiver operating characteristic (ROC) curves and calibration curves.

**FIGURE 2:**
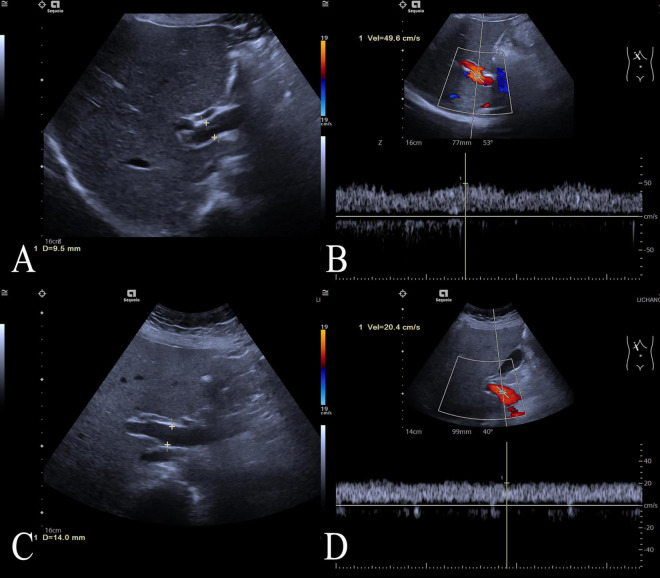
Two cases of portal vein measurement. Two cases of portal vein measurement using ultrasound imaging. **(A)** Ultrasound image of the control case showing a normal portal vein diameter measuring 9.5 mm, **(B)** and the case’s portal vein demonstrating a peak flow velocity of 49.6 cm/s; **(C)** Ultrasound image of an ALFP patient showing a wider portal vein diameter of 14.0 mm, **(D)** and the patient’s portal vein demonstrating a lower peak flow velocity of 20.4 cm/s.

The workflow of this study is shown in Figure 1. The study enrolled 135 individuals, split into a control group (*n* = 79) and a group of patients with acute fatty liver of pregnancy (AFLP, *n* = 56). The control group participants had an average age of 30 ± 4.6. Comparatively, the AFLP group exhibited a slightly higher average age of 31 ± 4.8, although statistical analysis revealed no significant differences between the groups. The training cohort and test cohort included 40 AFLPs and 56 healthy controls and 16 AFLPs and 23 healthy controls, respectively. The clinical characteristics of the training cohort and test cohort is shown in Table 1. Two cases of portal vein measurement is shown in Figure 2.

**TABLE 1:** The clinical characteristics of the acute fatty liver of pregnancy patients and healthy controls.

	Training cohort			Test cohort		
	**AFLP (*N* = 40)**	**Control (*N* = 56)**	***p***	**AFLP (*N* = 16)**	**Control (*N* = 23)**	***p***
Age	31.5 ± 5.1	29.9 ± 4.8	0.124	31.8 ± 4.0	30.0 ± 4.0	0.189
Height	161 ± 5.3	162 ± 6.1	0.761	161 ± 5.5	161 ± 6.3	0.882
Weight	75.3 ± 11.5	66.5 ± 6.9	< 0.001	71.5 ± 6.8	66.3 ± 7.9	0.035
BMI	28.9 ± 4.1	25.5 ± 2.7	< 0.001	27.7 ± 3.3	25.7 ± 2.3	0.045
Gestational age	36.4 ± 4.0	37.6 ± 2.7	0.100	37.9 ± 2.5	38.1 ± 3.1	0.815
Left lobe cranio-caudal diameter (cm)	6.9 ± 0.9	6.5 ± 0.6	0.015	6.7 ± 0.8	6.5 ± 0.6	0.400
Left lobe ventro-dorsal dimension (cm)	6.5 ± 0.8	6.2 ± 0.7	0.057	6.7 ± 0.7	6.4 ± 0.5	0.294
Right lobe ventro-dorsal dimension (cm)	11.7 ± 0.9	11.7 ± 0.8	0.915	11.6 ± 1.0	11.7 ± 0.7	0.857
Right lobe maximum dimension (cm)	13.3 ± 1.0	13.2 ± 0.9	0.430	13.2 ± 1.2	13.0 ± 0.6	0.609
Portal vein width (mm)	12.1 ± 1.2	10.5 ± 1.1	< 0.001	11.7 ± 1.3	10.2 ± 1.0	0.001
Portal vein flow velocity (cm/s)	23.2 ± 3.9	28.2 ± 4.5	< 0.001	22.2 ± 3.3	26.4 ± 4.2	0.002
Alanine aminotransferase (U/L)	17.1 ± 10.9	12.4 ± 7.2	0.020	14.3 ± 6.0	11.7 ± 9.8	0.309
Aspartate aminotransferase (U/L)	19.6 ± 9.4	17.3 ± 4.1	0.163	16.9 ± 6.6	16.1 ± 5.2	0.688
Total bilirubin (umol/L)	7.0 ± 1.8	7.4 ± 2.3	0.336	6.4 ± 2.0	6.9 ± 3.1	0.603
Total bile acids (umol/L)	3.6 ± 1.5	3.4 ± 1.4	0.486	4.3 ± 5.5	3.6 ± 1.2	0.646
Albumin (g/L)	37.8 ± 9.0	38.3 ± 9.6	0.806	38.1 ± 9.2	40.2 ± 11.3	0.516
Glucose (mmol/L)	5.3 ± 1.0	5.0 ± 0.9	0.207	5.3 ± 0.8	5.3 ± 0.9	0.950
Glycated hemoglobin (%)	4.9 ± 0.8	5.1 ± 0.5	0.134	5.1 ± 0.4	4.9 ± 0.8	0.462
Triglycerides (mmol/L)	5.2 ± 2.7	4.5 ± 3.1	0.248	4.7 ± 2.0	5.7 ± 3.03	0.233
Total cholesterol (mmol/L)	6.9 ± 1.6	7.1 ± 1.4	0.481	7.7 ± 1.2	7.4 ± 1.7	0.530
Low-density lipoprotein cholesterol (mmol/L)	3.1 ± 0.8	3.7 ± 1.0	0.002	3.5 ± 0.9	3.4 ± 1.1	0.642
High-density lipoprotein cholesterol (mmol/L)	2.0 ± 0.5	2.0 ± 0.3	0.585	2.0 ± 0.3	2.1 ± 0.4	0.572
Urea nitrogen (mmol/L)	3.8 ± 2.2	3.5 ± 1.4	0.569	4.9 ± 5.0	4.5 ± 2.7	0.770
Creatinine (umol/L)	42.9 ± 9.0	46.3 ± 10.2	0.094	44.1 ± 10.0	43.8 ± 10.0	0.943
Uric acid (umol/L)	206 ± 37.5	186 ± 38.8	0.013	204 ± 37.3	199 ± 40.3	0.747
C-reactive protein (mg/L)	43.1 ± 23.6	47.8 ± 23.4	0.331	42.9 ± 16.4	36.7 ± 15.2	0.238

AFLP, acute fatty liver of pregnancy.

### 3.1 Laboratory tests

The AFLP group exhibited elevated BMI compared to the control group (*p* < 0.001). The AFLP group exhibited elevated levels of liver enzymes compared to the control group, with alanine aminotransferase (ALT) averaging 16.3 U/L in the AFLP group versus 12.2 U/L in the control group (*p* = 0.011). There was a slight increase in total bile acids in the AFLP group (3.8 μmol/L) compared to the control group (3.5 μmol/L), though this difference was not statistically significant (*p* = 0.453). The groups exhibited similar total bilirubin, glucose, glycated hemoglobin levels, total cholesterol levels, HDL cholesterol and triglycerides. However, the LDL cholesterol was significantly lower in the AFLP group (3.2 μmol/L) compared to the control group (3.6 μmol/L, *p* = 0.025). Measures of kidney function including creatinine and urea nitrogen showed no significant differences between the groups. However, uric acid levels were significantly higher in the AFLP group (205 vs. 190 μmol/L in the control group, *p* = 0.022). Prothrombin time and albumin levels were similar across the groups, with no significant differences observed. The C-reactive protein levels, a marker of inflammation, were nearly identical between the groups, reinforcing the lack of significant inflammatory response difference in this context.

### 3.2 Doppler ultrasonography findings

The Doppler ultrasonography findings from the study provide detailed insights into the structural and functional characteristics of the liver and portal vein in both control and AFLP groups. Notably, the AFLP group exhibited a slightly larger left hepatic lobe cranio-caudal diameter, measuring an average of 6.9 cm compared to 6.5 cm in the control group, a difference that was statistically significant (*p* = 0.011). Although the left hepatic lobe ventro-dorsal dimension also trended larger in the AFLP group (6.6 vs. 6.3 cm, *p* = 0.029). However, right hepatic lobe measurements showed no differences between the two groups, indicating localized differences in liver enlargement.

**FIGURE 3:**
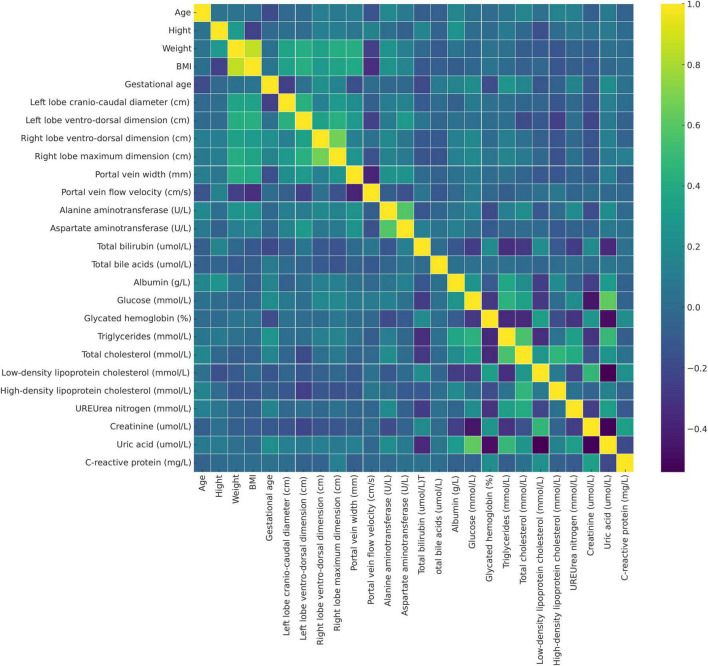
Heatmap displaying the correlation matrix of various clinical and Doppler ultrasonography parameters in the study population, including pregnant women with acute fatty liver of pregnancy (AFLP) and healthy controls. Each cell represents the correlation coefficient between two variables (purple, indicating negative correlation and yellow, indicating strong positive correlation). This heatmap helps identify potential interdependencies among variables and the relationship of these markers with AFLP pathology.

For changes in the portal vein characteristics, the AFLP group showed a significantly wider portal vein (12.0 mm compared to 10.4 mm in the control group, *p* < 0.001) and a lower portal vein flow velocity (22.9 cm/s compared to 27.7 cm/s, *p* < 0.001). These changes suggest a significant alteration in vascular dynamics in AFLP, likely reflecting hemodynamic adjustments or potential compensations in response to liver stress or dysfunction. A heatmap displaying the correlation matrix of various clinical and Doppler ultrasonography parameters is shown in Figure 3.

### 3.3 Performance evaluation and validation of the nomogram model

**FIGURE 4:**
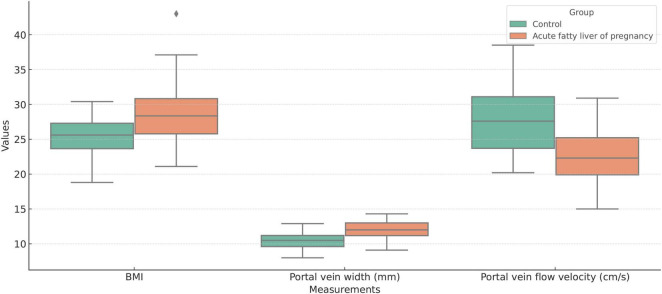
Boxplot comparison of Body Mass Index (BMI), portal vein width (mm), and portal vein flow velocity (cm/s) between the acute fatty liver of pregnancy (AFLP) group and healthy control group. The AFLP group (orange) shows higher BMI and wider portal vein diameter compared to the control group (green). Portal vein flow velocity is significantly lower in the AFLP group, indicating alterations in vascular dynamics associated with liver dysfunction. Each box represents the interquartile range (IQR), the central line indicates the median, and whiskers denote variability outside the upper and lower quartiles. Outliers are displayed as individual points.

Multivariate logistic regression analysis showed that body mass index (BMI), portal vein width, and portal vein flow velocity were the key parameters in differentiating AFLP from healthy control cases. A nomogram was built by integrating these three parameters (Figure 4).

**FIGURE 5:**
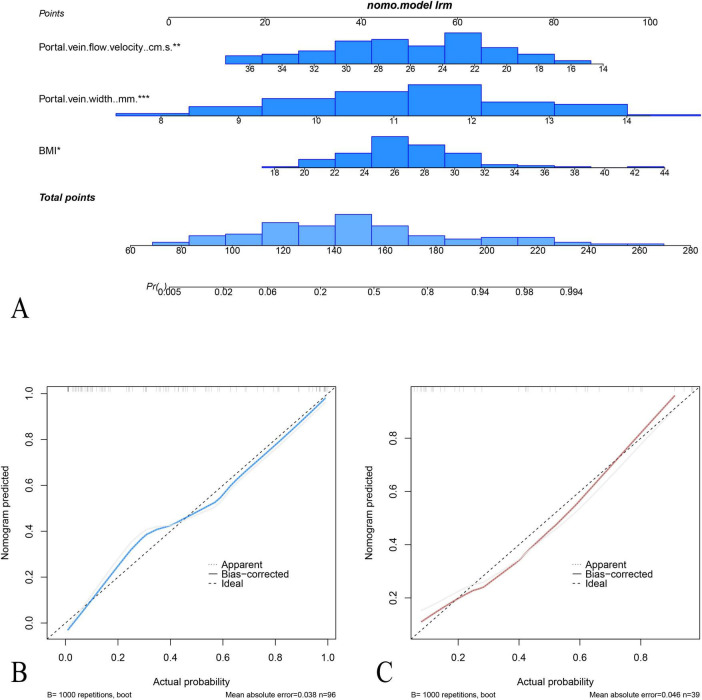
Nomogram and calibration curves for the prediction of acute fatty liver of pregnancy (AFLP). **(A)** The nomogram integrates three predictors: portal vein flow velocity, portal vein width, and BMI, to calculate the probability of AFLP. Each predictor is assigned a point value based on its contribution, and the total points determine the estimated probability of AFLP. Calibration plots for the nomogram in the training cohort **(B)** and test cohort **(C)** display the agreement between predicted and observed probabilities of AFLP. In both cohorts, the nomogram shows good calibration, as indicated by close alignment between the ideal and bias-corrected lines.

The area under the receiver operating characteristic (ROC) curves (AUC) of the nomogram model were 0.88 (95% confidence interval [CI]: 0.82–0.95) with specificity, sensitivity, negative predictive value, and positive predictive value of 67.8, 95.0, 95.0, and 67.8, respectively, in the training cohort and 0.86 (95% CI: 0.72–0.98) and 86.9, 75.0, 83.3, and 80.0 in the test cohort, respectively. The calibration curves demonstrated good agreement between the predicted and observed probability for predicting AFLP (Figure 5). The ROC curves of the BMI, Portal vein width, portal vein flow velocity and the nomogram is shown in (Supplementary Figure 1).

## 4 Discussion

Our study sought to delineate the relationship between AFLP and portal vein flow dynamics during pregnancy. We observed significant physiological adaptations that manifest in distinct vascular and hepatic changes during this critical period. A key observation is the gestational age distribution, predominantly in the late third trimester, which aligns with the critical period of fetal development and maternal physiological changes. The results suggest that AFLP may significantly impact portal velocity during pregnancy.

The significant differences in weight and BMI between the AFLP and control groups indicate their potential role in influencing portal vein dynamics. BMI, in particular, emerged as a key predictor in our nomogram model, reflecting its possible contribution to the pathophysiology of AFLP. Increased BMI is associated with metabolic and vascular changes, which may exacerbate the risk of hepatic dysfunction during pregnancy.

The reduction in portal vein flow velocity and alterations in hepatic dimensions noted in our AFLP group align with findings from similar studies, underscoring the sensitivity of Doppler ultrasonography in detecting hepatic dysfunction (9). Portal vein flow velocities is reported increased in pregnancy in late pregnancy (10). However, decreased portal vein flow velocities with the resultant hepatic arterial flow and velocity could be found in cirrhosis and portal hypertension (11). A study reported a decrease in portal vein flow velocities associated with increased liver strain and potential gestational complications. Unlike traditional liver function tests, which may not fully capture the onset of liver-related pregnancy complications, Doppler measurements provide a dynamic insight into real-time physiological shifts (12). In addition to Doppler ultrasonography, emerging research has explored the role of other imaging modalities in the diagnosis and management of AFLP (13). A study investigated the use of MRI in assessing liver function and disease severity in pregnant women with AFLP, demonstrating its potential as a complementary tool to Doppler ultrasonography (14). Similarly, a recent meta-analysis evaluated the diagnostic accuracy of various imaging techniques, including Doppler ultrasonography, MRI, and CT, in detecting AFLP, highlighting the importance of multimodal imaging approaches in clinical practice (15).

Moreover, our results corroborate previous studies, that found the changes in the hepatic vein waveform patterns from triphasic to biphasic during pregnancy can serve as early indicators of liver stress before clinical symptoms manifest (16). These studies collectively suggest that routine Doppler ultrasonography could be pivotal in the prenatal screening regimen, especially in populations at higher risk of AFLP. A study investigated hepatic morphology using ultrasound imaging and found distinct changes in liver dimensions, including the left hepatic lobe, in non-alcoholic fatty liver disease patients (17). Recent studies have focused on specific hepatic morphological changes in AFLP (18). Shekarriz-Foumani et al. (19) examined left hepatic lobe craniocaudal diameter and left hepatic lobe anteroposterior diameter in pregnant women with AFLP, revealing significant alterations compared to healthy controls.

The clinical implications of our findings are substantial. The identification of decreased portal vein flow velocity as a potential early marker for AFLP allows for the stratification of patients based on risk and can guide the intensity of monitoring and early intervention strategies. Furthermore, our study suggests a need to reconsider the reliance on traditional liver function tests during pregnancy. Given the physiological liver changes during pregnancy, these tests may not always be reliable indicators of true liver health. Incorporating Doppler ultrasonography measurements could thus refine our diagnostic algorithms, ensuring more timely and accurate detection of complications like AFLP.

While our study used logistic regression, the potential for machine learning (ML) models, particularly ensemble methods like random forests or gradient boosting, to capture non-linear relationships among variables (20). These models might improve predictive accuracy by modeling complex interactions without pre-specifying their form. Future work should compare ML models with traditional approaches to assess their ability to refine diagnostic strategies for AFLP. In this study, several factors were identified related to portal vein flow velocity and its association with AFLP, the causal relationships between these factors and the outcome remain largely unknown. The observed correlations in our model do not imply causality, and it is important to recognize that other unmeasured variables or confounders may also contribute to the outcomes (21). To better understand the causal pathways, future studies should consider employing causal inference methods, which could provide deeper insights into the directionality and strength of these relationships. The application of causal models could have significant implications for clinical decision-making, particularly in terms of early diagnosis and intervention for AFLP. Further research with a focus on causal inference is needed to establish definitive cause-and-effect relationships, which will enhance the clinical utility of portal vein flow velocity as a diagnostic marker for AFLP.

While our findings are promising, they necessitate validation through larger, multicentric studies that would allow for a broader generalization of the results. Future research should also aim to establish a comprehensive set of Doppler ultrasonography criteria, including standardized thresholds for portal vein flow velocity that can be seamlessly integrated into clinical practice. Additionally, longitudinal studies spanning the entirety of pregnancy could provide deeper insights into the trajectory of portal vein and liver function changes, offering a timeline for potential intervention.

## 5 Conclusion

In summary, our study reinforces the potential of portal vein flow velocity measurements as predictive markers of liver dysfunction during pregnancy. The integration of these parameters into routine prenatal screening, particularly for women at risk of AFLP, could significantly enhance clinical outcomes through earlier diagnosis and tailored management strategies. Further research is essential to refine these findings and fully translate them into practice, ensuring that pregnant women receive the most informed and effective care possible.

## Data Availability

The raw data supporting the conclusions of this article will be made available by the authors, without undue reservation.

## Footnotes

1
https://www.r-project.org/

## Supplementary material

Supplementary Figure 1 The ROC curves of the BMI, portal vein width, portal vein flow velocity and the nomogram (combined features).
