# Supplementary figures and images for: Reduced portal vein blood flow velocity in acute fatty liver of pregnancy
Source: Front Med (Lausanne). 2024 Dec 23;11:1506340. doi: 10.3389/fmed.2024.1506340 (PMC11701050; doi:10.3389/fmed.2024.1506340)

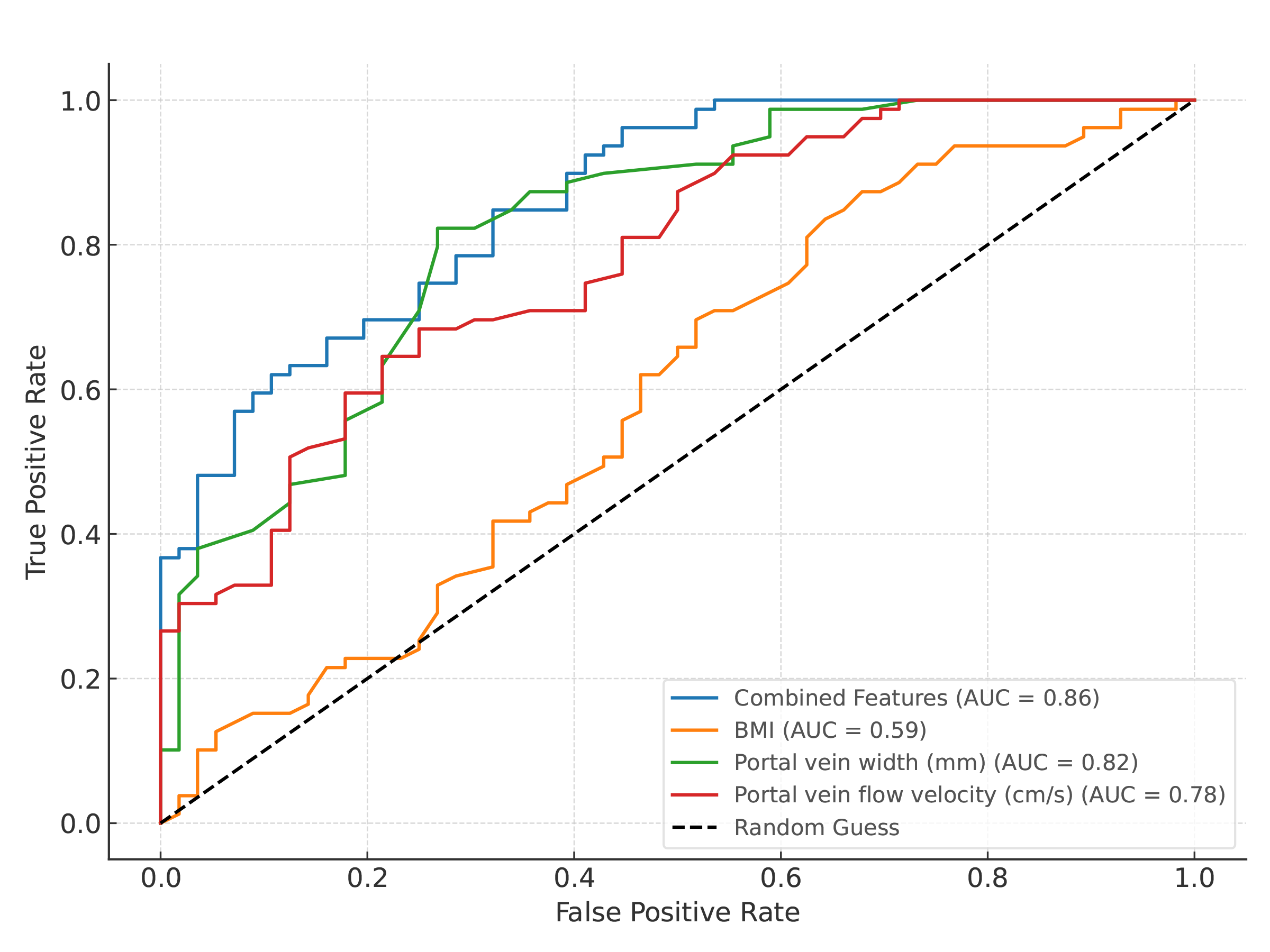

Supplement: Supplementary Figure 1 — The ROC curves of the BMI, portal vein width, portal vein flow velocity and the nomogram (combined features). [file Image_1.tiff]
